# RUNX3 overexpression inhibits normal human erythroid development

**DOI:** 10.1038/s41598-022-05371-z

**Published:** 2022-01-24

**Authors:** Ana Catarina Menezes, Christabel Dixon, Anna Scholz, Rachael Nicholson, Adam Leckenby, Aleksandra Azevedo, Sarah Baker, Amanda F. Gilkes, Sara Davies, Richard L. Darley, Alex Tonks

**Affiliations:** 1 Division of Cancer & Genetics, Department of Haematology, School of Medicine, Cardiff University, Cardiff, Wales CF14 4XN UK; 2 Cardiff Experimental Cancer Medicine Centre (ECMC), School of Medicine, Cardiff University, Cardiff, CF14 4XN UK

**Keywords:** Haematological cancer, Leukaemia

## Abstract

RUNX proteins belong to a family of transcription factors essential for cellular proliferation, differentiation, and apoptosis with emerging data implicating RUNX3 in haematopoiesis and haematological malignancies. Here we show that RUNX3 plays an important regulatory role in normal human erythropoiesis. The impact of altering RUNX3 expression on erythropoiesis was determined by transducing human CD34^+^ cells with RUNX3 overexpression or shRNA knockdown vectors. Analysis of *RUNX3* mRNA expression showed that *RUNX3* levels decreased during erythropoiesis. Functionally, RUNX3 overexpression had a modest impact on early erythroid growth and development. However, in late-stage erythroid development, RUNX3 promoted growth and inhibited terminal differentiation with RUNX3 overexpressing cells exhibiting lower expression of glycophorin A, greater cell size and less differentiated morphology. These results suggest that suppression of RUNX3 is required for normal erythropoiesis. Overexpression of RUNX3 increased colony formation in liquid culture whilst, corresponding RUNX3 knockdown suppressed colony formation but otherwise had little impact. This study demonstrates that the downregulation of RUNX3 observed in normal human erythropoiesis is important in promoting the terminal stages of erythroid development and may further our understanding of the role of this transcription factor in haematological malignancies.

## Introduction

Transcription factors play an important role in the establishment of haematopoietic lineages by regulating not only the survival and proliferation of haematopoietic stem and progenitor cells (HSPC), but also cell fate decisions and differentiation^1^. Their disruption can lead to changes in haematopoietic differentiation and the subsequent development of haematopoietic malignancies. RUNX proteins are a family of transcription factors (RUNX1, 2 and 3) that participate in important developmental processes: RUNX1 is essential for definitive haematopoiesis^2,3^; RUNX2 is involved in skeletal development^4,5^; and RUNX3 is essential for neurogenesis^6,7^, T cell development^8,9^ and gastric epithelium growth^10^. Whilst there are several studies describing the central role of RUNX1 in haematopoiesis, little is known regarding the role of RUNX3 in human haematopoiesis.

Emerging data has supported an important role for RUNX3, the evolutionary founder of the mammalian RUNX family, in murine haematopoiesis^11^. RUNX3 is highly expressed in HSPC and its conditional knockout in aged mice causes a mild HSPC expansion and myeloid proliferation, partially phenocopying *RUNX1* conditional knockout mice^12^. Indeed, conditional loss of *RUNX1* in adult mice was previously shown to induce a transient expansion of haematopoietic stem cells followed by their subsequent exhaustion^13,14^. An interplay between RUNX1 and RUNX3 has been found in a RUNX1/RUNX3 double knockout model, with mice dying as a result of either bone marrow failure or a myeloproliferative disorder^15^. Furthermore, RUNX3 overexpression was recently shown to facilitate the development of a myelodysplastic syndrome in TET2-deficient mice, characterised by a disruption of cancer-related pathways and RUNX1-mediated haematopoiesis^16^. Interestingly, RUNX3 overexpression is considered an independent prognostic factor associated with worse event-free survival in childhood AML^17^. On the other hand, RUNX3 expression was found to be downregulated in prognostically favourable core binding factor (CBF) AML involving RUNX1-ETO and CBFβ-MYH11 fusion proteins^17^. Previous studies have shown that RUNX1-ETO expression as a single abnormality in human HSPC blocks erythroid differentiation and promotes self-renewal of HSPC^18,19^. A recent study showed that HSPC from elderly patients with unexplained anaemia present a greater reduction in *RUNX3* expression and yield fewer erythroid colonies compared to non-anaemic progenitors^20^. Considering the growing evidence for an important role in haematopoiesis and haematological malignancies, this study sought to establish the role of RUNX3 expression on normal human erythroid development.

## Results

### Expression of RUNX3 declines during human terminal erythroid maturation

**Figure 1:**
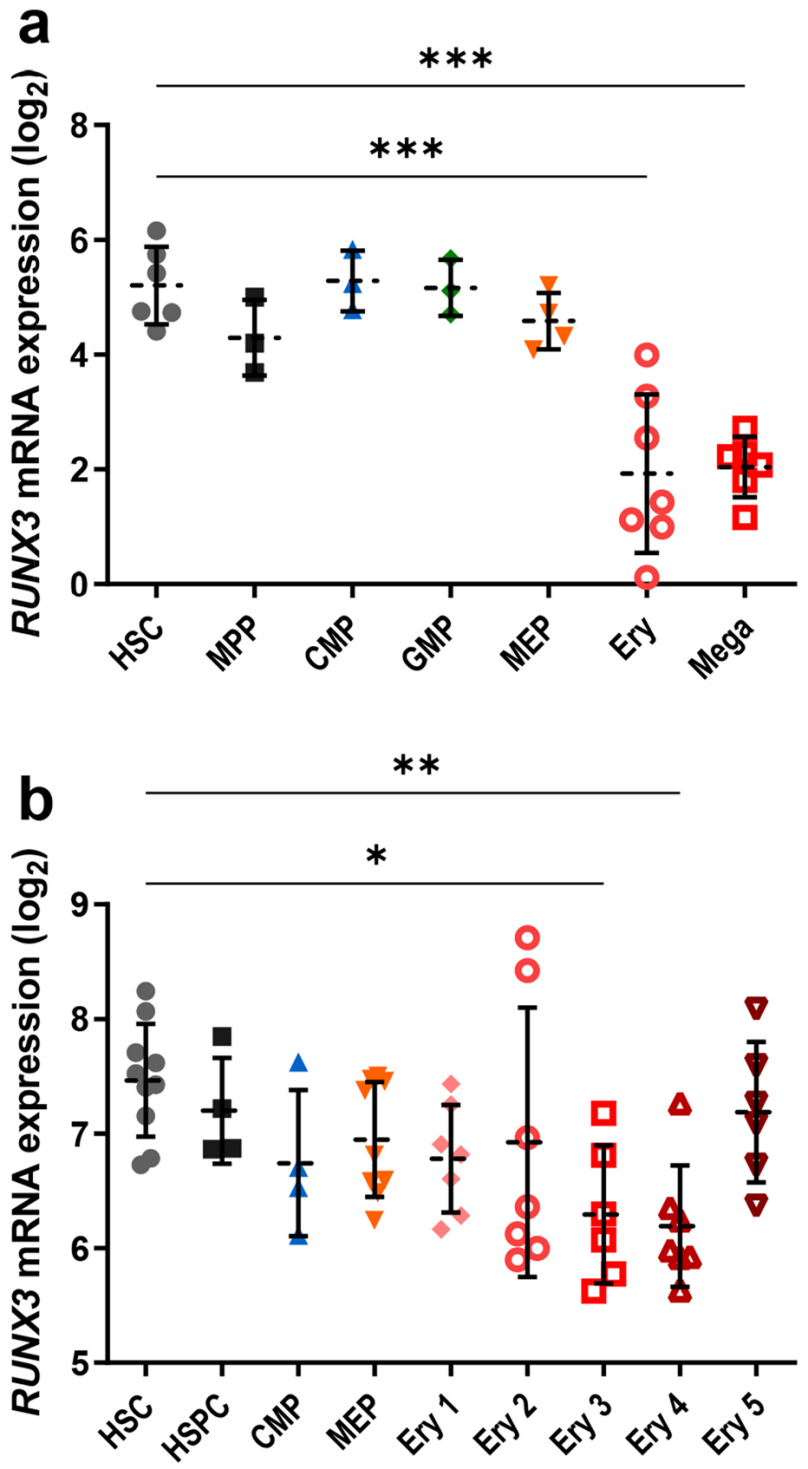
*RUNX3* mRNA expression levels decrease during normal human erythropoiesis. (**a**) *RUNX3* mRNA expression (log_2_ transformed) within distinct human haematopoietic cell subsets. MPP—Multipotent progenitor; GMP—Granulocyte/monocyte progenitor; Ery—Erythroblast; Mega—Megakaryocytic cell CD34^−^CD41^+^. RNA-sequencing data obtained from the BLUEPRINT study^43^. Data indicate mean ± 1SD (n ≥ 3). Statistical analysis was performed using ANOVA with Tukey’s multiple comparisons test, ***p < 0.001 vs HSC. (**b**) *RUNX3* mRNA expression (log_2_ transformed) in distinct haematopoietic cell subsets based on cell surface marker expression. HSC—Haematopoietic stem cell CD133^+^CD34^dim^; HSPC—Haematopoietic stem progenitor cell CD38^−^CD34^+^; CMP—Common myeloid progenitor; MEP—Megakaryocyte/erythroid progenitor; Ery 1—Erythroid CD34^+^CD71^+^GPA^−^; Ery 2—Erythroid CD34^−^CD71^+^GPA^−^; Ery 3—Erythroid CD34^−^CD71^+^GPA^+^; Ery 4—Erythroid CD34^−^CD71^low^GPA^+^; Ery 5—Erythroid CD34^−^CD71^−^GPA^+^. Data obtained from GSE24759 using 204197_s_at probeset^42^. Data indicate mean ± 1SD (n ≥ 4). Statistical analysis was performed using one-way ANOVA with Tukey’s multiple comparisons test, *p < 0.05; **p < 0.01 vs HSC*.*

To elucidate the potential role of RUNX3 expression in normal human erythroid development, expression of *RUNX3* was assessed in different haematopoietic cell subsets. *RUNX3* mRNA is expressed within the HSC compartment and myeloid progenitor cells whereas erythroblasts display comparatively lower levels (Fig. 1a). *RUNX3* expression reduces further as erythroblasts differentiate into mature erythroid cells (Fig. 1b) indicating that suppression of RUNX3 might play a role in the terminal differentiation of erythroid cells.

### Overexpression of RUNX3 suppresses human erythroid development

**Figure 2:**
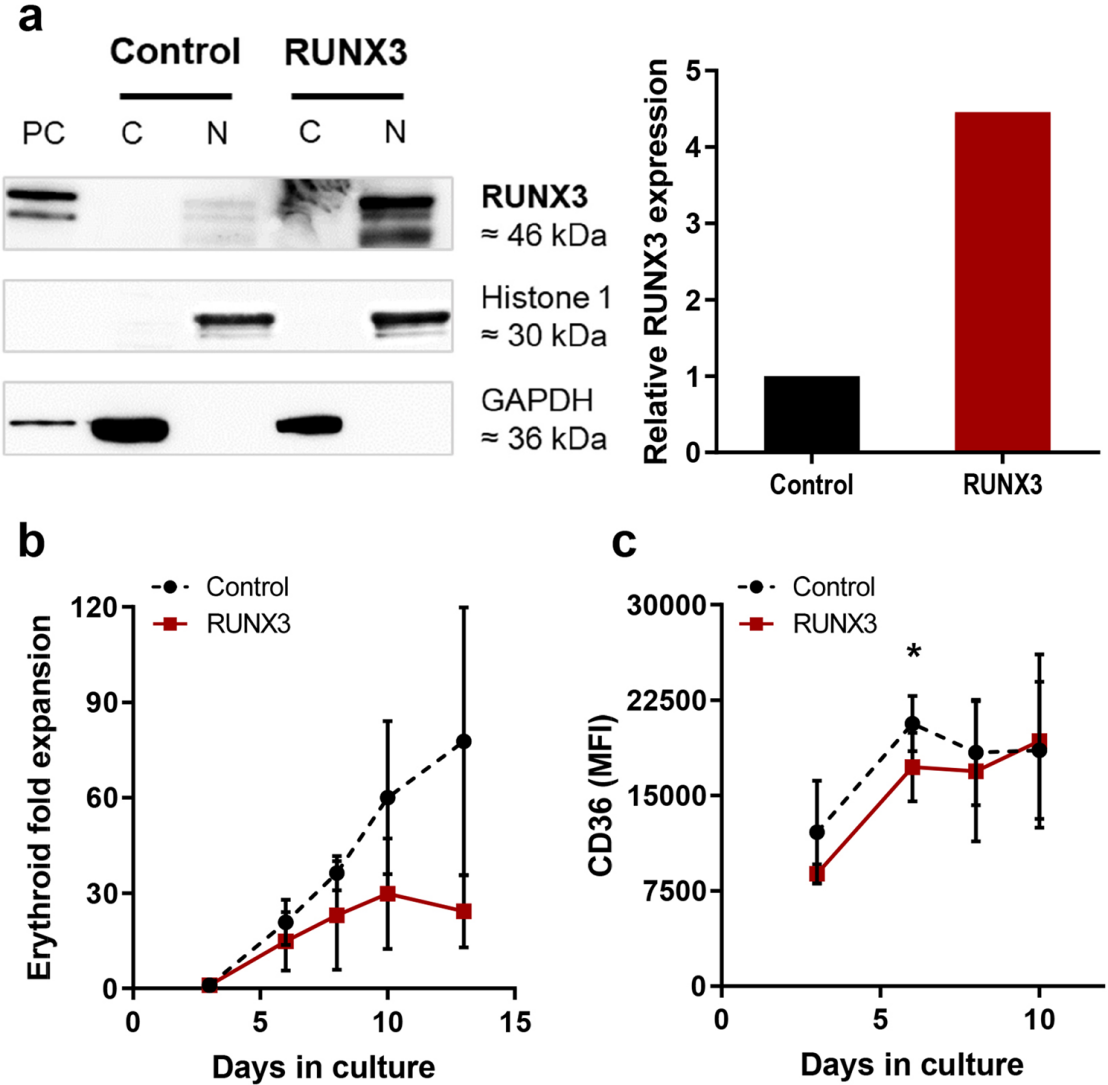
RUNX3 overexpression inhibits CD36 upregulation and proliferation in HSPC during erythropoieitin (EPO) independent erythroid differentiation. (**a**) *Left Panel:* Western blot showing RUNX3 protein levels in the cytosol and nucleus of control and RUNX3 CD34^+^ HSPC (day 6 of culture). Phoenix packaging cells overexpressing RUNX3 were used as a positive control and Histone 1/GAPDH were used as loading controls. PC—Positive control; C—Cytosol; N—Nucleus. Same membranes were reprobed for Histone H1 and GADPH. Full length blots are included in Supplemental Fig. S3. *Right panel*: Relative RUNX3 nuclear expression in control and RUNX3 HSPC cells (day 6 of culture) normalised to control. (**b**) Cumulative fold expansion of CD13^low^CD36^high^ erythroid committed cells during the EPO independent phase of growth. Data indicate mean ± 1SD (n ≥ 3). (**c**) Summary data of CD36 mean fluorescence intensity (MFI) in CD13^low^CD36^high^ erythroid committed cells over time. Data indicate mean ± 1SD (n ≥ 3). Significant difference of RUNX3-expressing cells from controls was analysed by paired t-test, *p < 0.05.

To determine whether suppression of RUNX3 is functionally important to normal erythroid development, human cord blood derived CD34^+^ HSPC were stably transduced with a vector co-expressing RUNX3 and DsRed (Supplemental Fig. S1). Cultures were subsequently enriched for transduced erythroid cells (DsRed^+^CD13^low^) by FACS to aid the analysis of the retrovirally transduced erythroid population (CD13^low^CD36^high^) (Supplemental Fig. S2). Overexpression of RUNX3 protein was validated by western blot, showing a 4.4-fold increase in RUNX3 nuclear levels compared to control cells (Fig. 2a and Supplemental Fig. S3).

Erythroid differentiation can be divided into an early developmental stage which occurs independently of EPO and a late developmental stage strictly dependent on this cytokine^21^. The growth and differentiation of the erythroid committed population (CD13^low^CD36^high^) was first assessed by culturing cells in the absence of EPO. We found that while the growth of control cultures continued to day 13, the growth of RUNX3 cultures ceased by day 10 displaying a four fold reduction in proliferative capacity by day 13 compared to controls (Fig. 2b).

Phenotypic changes associated with early erythropoiesis are characterized by an increase of CD36 expression with a simultaneous loss of CD34^22^. RUNX3 overexpression delayed upregulation of CD36 (Fig. 2c) though no significant impact on CD34 expression was observed (data not shown). Together, these data suggest that overexpression of RUNX3 in human HSPC suppresses the growth and early development of erythroid progenitors in the absence of EPO.

**Figure 3:**
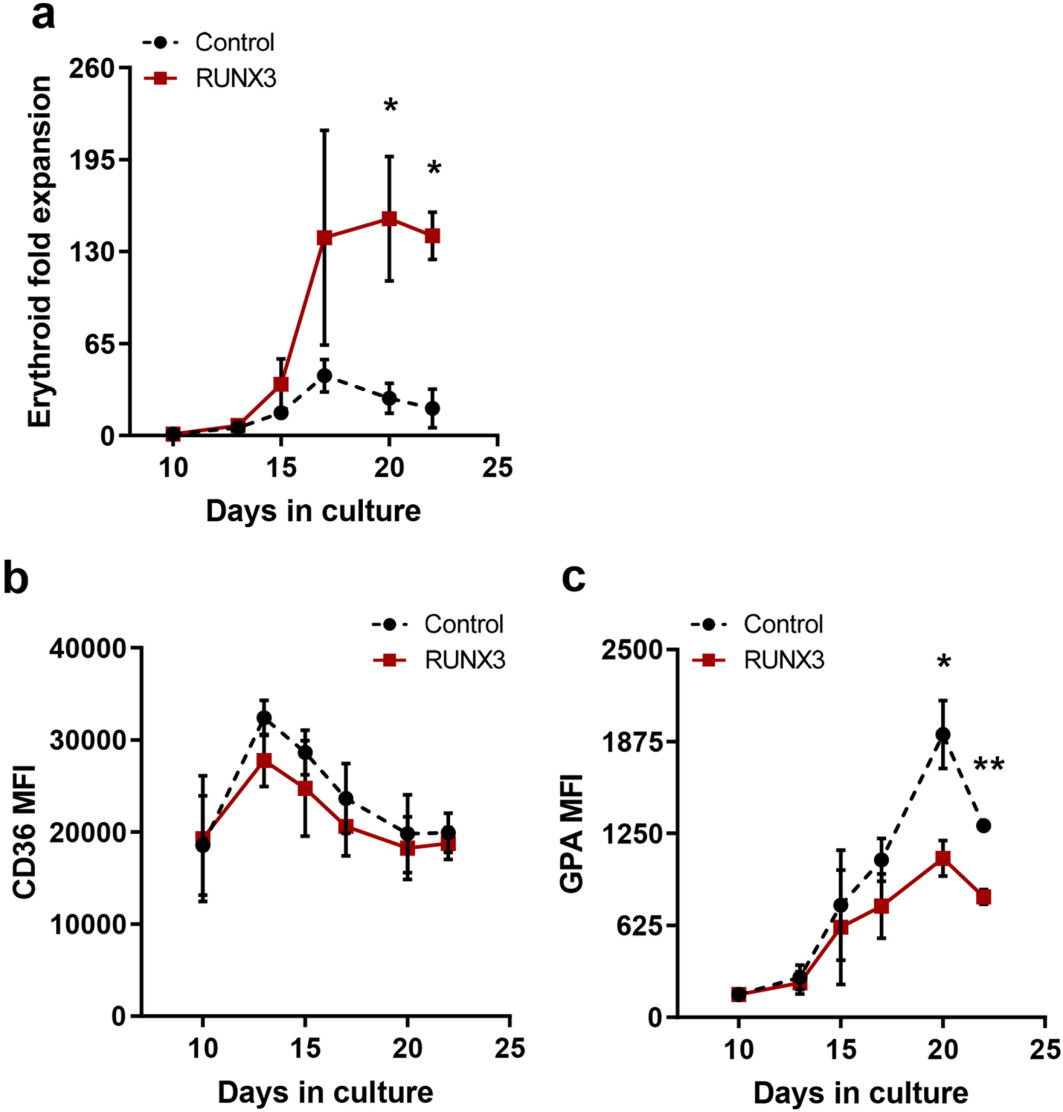
RUNX3 overexpression promotes proliferation and supresses GPA expression during the EPO dependent phase of differentiation. (**a**) Cumulative fold expansion of CD13^low^CD36^high^ erythroid committed cells during the EPO dependent phase of growth. HSPC were initially cultured with IL-3, IL-6 and SCF for 10 days followed by addition of EPO at day 10. (**b**) Summary data of CD36 and (**c**) GPA MFI in CD13^low^CD36^high^ erythroid committed cells over time. Data indicate mean ± 1SD (≥ 4). Significant difference of RUNX3-expressing cells from controls was analysed by paired t-test, *p < 0.05, **p < 0.01.

**Figure 4:**
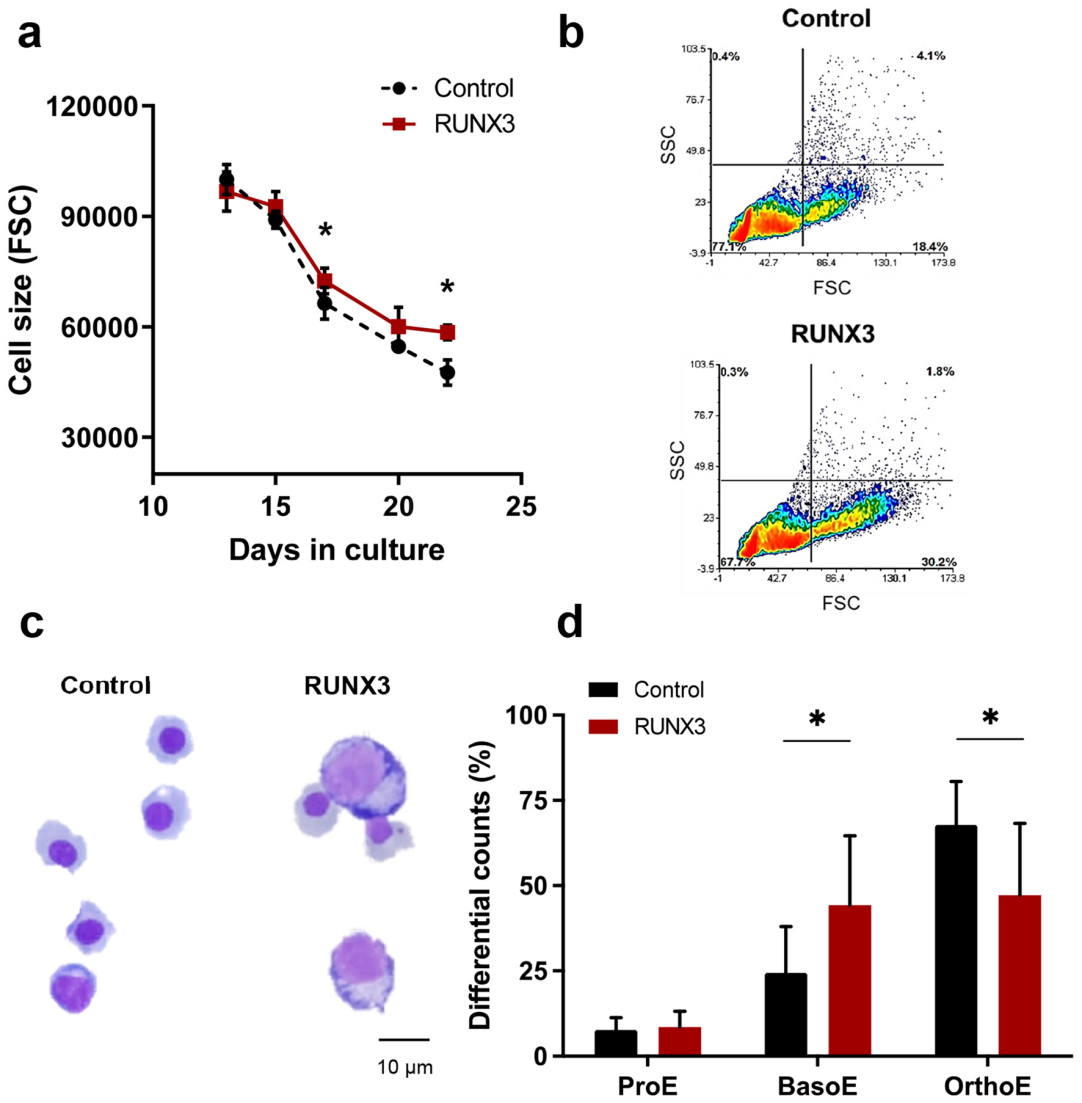
RUNX3 overexpression inhibits terminal erythroid differentiation. (**a**) Summary plot showing cell size assessed by flow cytometry (forward scatter, FSC). HSPC were initially cultured with IL-3, IL-6 and SCF for 10 days followed by addition of EPO at day 10. (**b**) Representative bivariate density plots of side scatter (SSC) *vs* FSC in control and RUNX3 cultures on day 22 of erythroid development. (**c**) Control and RUNX3-expressing cells analysed on day 20 of differentiation with May-Grünwald-Giemsa. (**d**) Differential morphology counts of cells categorised into ProE (proerythroblasts), BasoE (basophilic erythroblasts) and OrthoE (orthochromatic erythroblasts). Data indicate mean ± 1SD (n = 5). Significant difference of RUNX3-expressing cells from controls was analysed by paired t-test, *p < 0.05.

The effects of RUNX3 overexpression on the EPO dependent phase of erythroid development were subsequently analysed. In the presence of EPO, erythroid progenitors re-enter cell cycle and upregulate glycophorin A (GPA). Subsequently, they undergo maturation-associated growth arrest accompanied by reduction of cell size and of CD36 expression^18,23–25^. In the presence of EPO, RUNX3-overexpressing erythroid cells showed enhanced proliferation compared to controls (7.0-fold by day 20; Fig. 3a). Developmentally, both cultures showed a decrease in CD36 expression as cells matured (Fig. 3b and Supplemental Fig. S4). However, RUNX3 overexpression significantly suppressed GPA expression (1.8-fold on day 20 Fig. 3c and Supplemental Fig. S5), implying that terminal differentiation was inhibited. In support of this, RUNX3 erythroid cells showed a consistently higher forward scatter (FSC) compared to control (Fig. 4a,b), suggesting that RUNX3 cells were significantly larger than control cells. Furthermore, morphological analysis demonstrated that while control cells were predominantly in a late stage of erythroid differentiation (OrthoE; orthochromatic erythroblast), RUNX3 overexpression reduced the number of cells with orthochromatic erythroblast morphology (Fig. 4c,d). Taken together, these data suggest that RUNX3 overexpression suppresses terminal erythroid differentiation.

**Figure 5:**
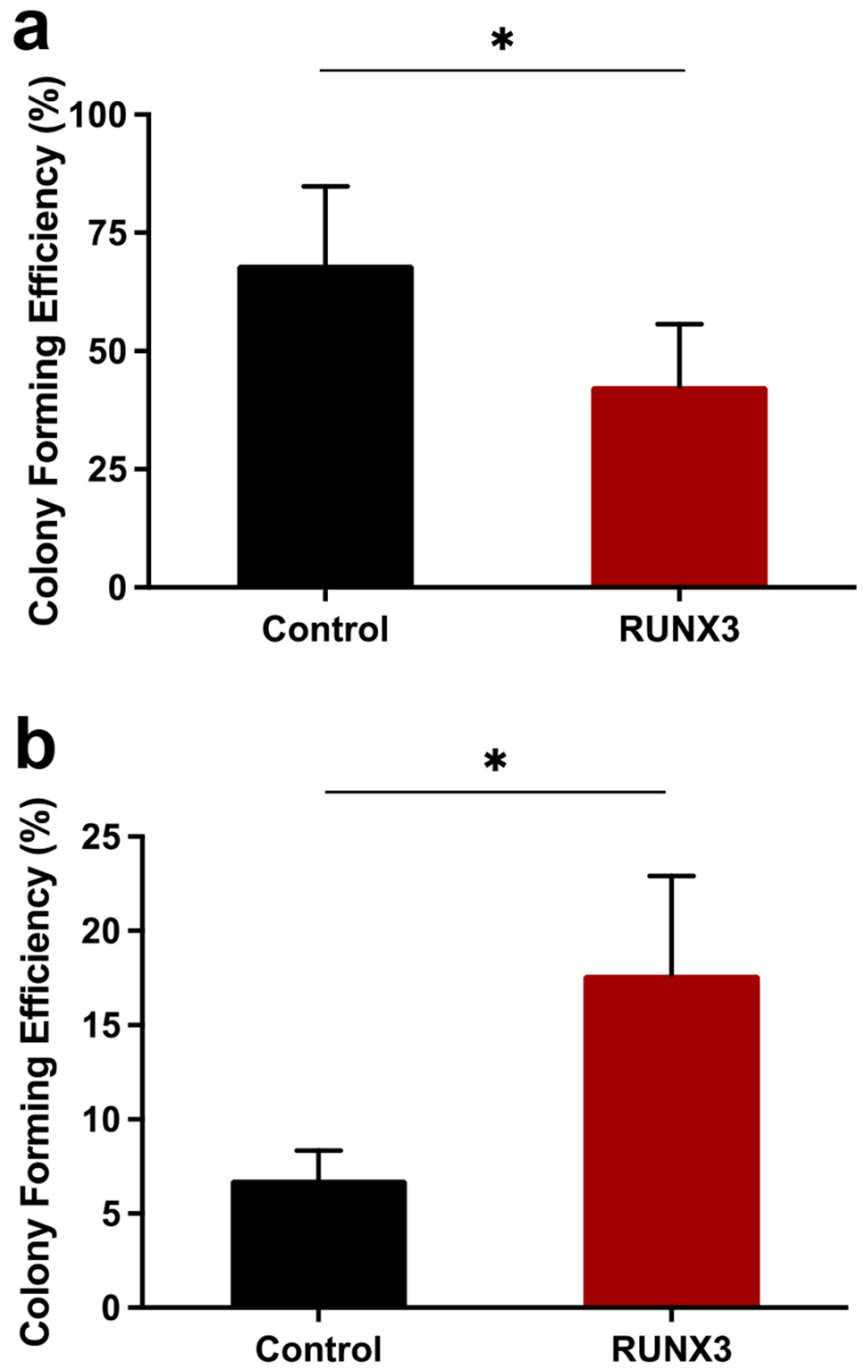
RUNX3 overexpression inhibits erythroid colony formation and increases colony formation in replating assays. (**a**) Erythroid colony forming efficiency of sorted DsRed^+^CD13^low^ control and RUNX3-expressing cultures following 7 days of growth in erythroid supporting growth medium containing IL-3, IL-6, SCF and EPO. Data indicate mean ± 1SD (n ≥ 3). No differences were observed in cluster formation between control and RUNX3 cultures (data not shown). (**b**) Self-renewal potential, assessed by a single replating round of control and RUNX3 cultures in the same conditions as previously. Following the initial 7 days of growth, erythroid colonies were counted, harvested and cells were replated in erythroid supporting growth medium containing IL-3, IL-6, SCF and EPO. Replating #1 data indicate mean ± 1SD (n = 3). Significant difference of RUNX3-expressing cells from controls was analysed by paired t-test, *p < 0.05. Replating #2 data was obtained from a single experiment.

We next determined the effects of RUNX3 overexpression on erythroid colony forming capacity and self-renewal under clonal conditions. RUNX3 overexpression resulted in a significant reduction in colony forming ability by 1.7-fold compared with control (Fig. 5a). To gauge the impact of RUNX3 overexpression on self-renewal potential we carried out serial replating of colony forming cells. RUNX3 overexpressing cells were able to form 2.7-fold more erythroid colonies than controls upon replating (Fig. 5b). These results indicate that while expression of RUNX3 impairs erythroid colony formation, these cells have a higher self-renewal potential consistent with the inhibition of differentiation observed above.

### Knockdown of RUNX3 expression impairs the colony forming efficiency of erythroid cells

**Figure 6:**
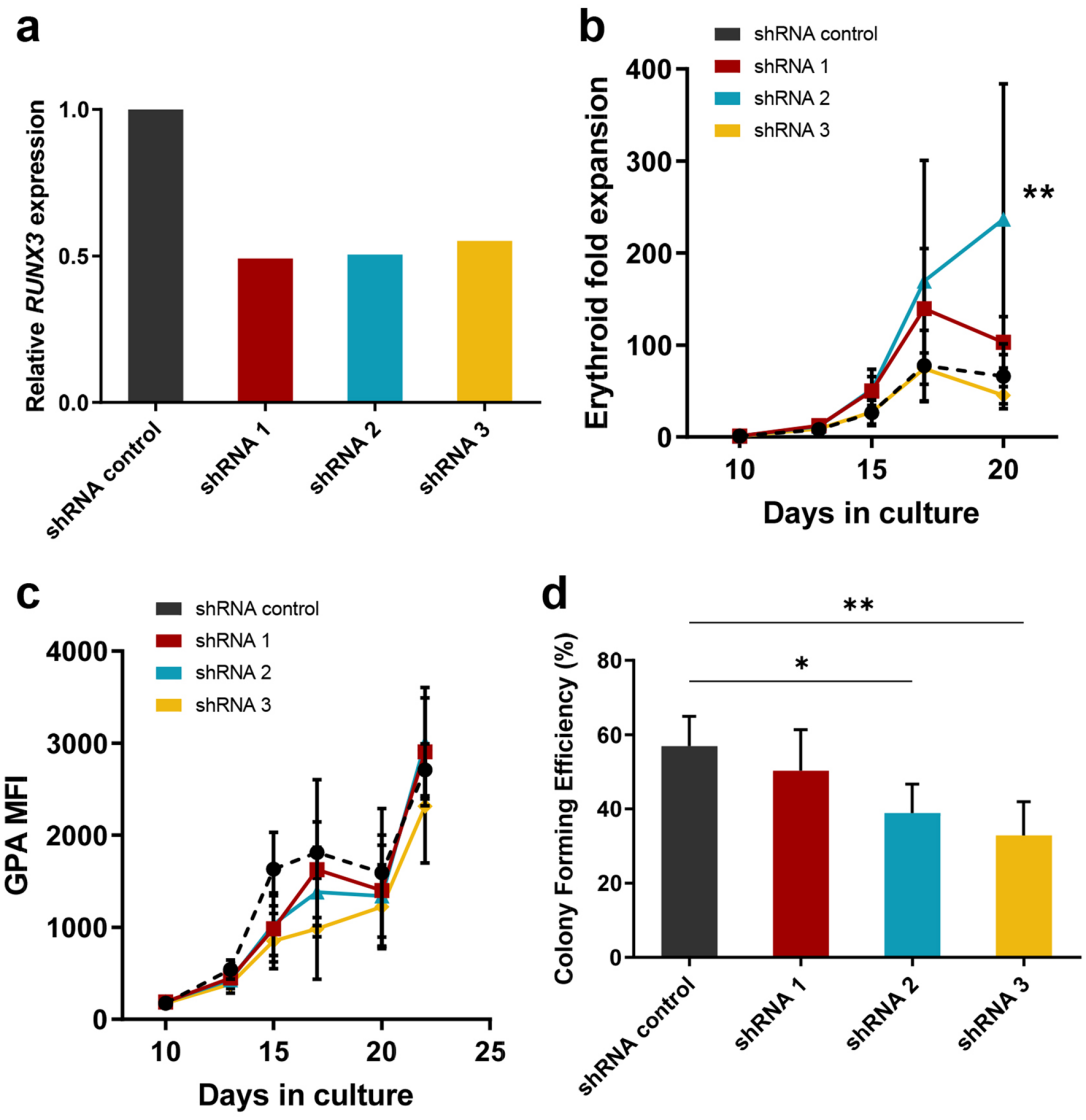
Knockdown of RUNX3 impairs erythroid colony formation efficiency, but not erythroid growth and development in bulk liquid culture. (**a**) qRT-PCR analysis of *RUNX3* mRNA levels in shRNA control and RUNX3 KD HSPC sorted for GFP positivity on day 3 of culture (n = 1). *GAPDH* was used as endogenous control. Relative expression calculated using the Comparative CT (ΔΔC_T_) method. (**b**) Cumulative expansion of erythroid progenitors in shRNA control and RUNX3 KD cultures during the EPO dependent phase of growth. HSPC were initially cultured with IL-3, IL-6 and SCF for 10 days followed by addition of EPO at day 10. Data indicate mean ± 1SD (n ≥ 3). Significant differences were analysed by one-way ANOVA using Tukey’s multiple comparisons test, **p < 0.01. (**c**) Summary data showing GPA expression (MFI) in GFP^+^CD13^low^CD36^high^ cells over time. Data indicate mean ± 1SD (n ≥ 3). (**d**) Erythroid colony forming efficiency of shRNA control and RUNX3 KD cultures after 7 days of growth in liquid culture. Data indicate mean ± 1SD (n ≥ 4). Significant differences were analysed by one-way ANOVA using Tukey’s multiple comparisons test, *p < 0.05, **p < 0.01.

We next sought to examine the effects of reducing endogenous levels of RUNX3 on human erythroid development. Lentiviral vectors encoding different RUNX3 shRNA were employed for this study (Supplemental Fig. S1). The knockdown (KD) of RUNX3 was validated by qRT-PCR and western blot in HSPC and OCI-AML-5 cells, respectively (Fig. 6a and Supplemental Fig. S6A). RUNX3 expression was reduced by approximately 50% for all three independent shRNA clones in comparison with control HSPC. In OCI-AML-5 cells, RUNX3 shRNA 1 had the strongest effect in reducing RUNX3 protein levels. During the EPO independent phase of development, RUNX3 KD impaired the growth (3.5-fold for shRNA 1, Supplemental Fig. S6B) of erythroid committed cells. Upon addition of EPO to the culture medium, no overall significance was observed in erythroid growth for RUNX3 KD cells, apart from shRNA 2 which could be attributable to off-target effects (Fig. 6b).

Developmentally, reduced RUNX3 expression induced a transient delay in GPA upregulation (Fig. 6c); however, cell size and morphology were unaffected by RUNX3 KD (Supplemental Fig. S6C,D). While RUNX3 KD impaired erythroid colony forming efficiency by 15–41% (Fig. 6d), self-renewal potential as scored by colony replating was not significantly affected, possibly due to selection bias for colonies with poorer RUNX3 KD in the replating round (Supplemental Fig. S7A). Taken together, reduced expression of RUNX3 impairs the colony forming ability of erythroid cells compared to controls but fails to induce similar effects on erythroid growth and development in bulk liquid culture. This suggests that RUNX3 KD impaired survival under clonal conditions, an observation supported by the fact that cluster formation was similarly impacted (Supplemental Fig. S7B). Overall, and in contrast to the effect observed in the overexpression studies, KD of RUNX3 had only minor consequences on erythroid development, with the caveat that we were unable to generate high efficiency RUNX3 KD in these cells.

## Discussion

RUNX3 (located at 1p36, a chromosomal region often deleted in several types of cancer) has a major role in the development of gastro-intestinal tract, neurogenesis and thymopoiesis^17,26^. Whilst this transcription factor has also been shown to be crucial during haematopoiesis in non-human models, there remains a paucity of studies regarding its role in normal human haematopoiesis. This study investigated the expression of RUNX3 and its role on human erythroid development using a normal human primary cell haematopoietic model.

In human cord blood derived haematopoietic cells, *RUNX3* mRNA expression levels gradually decreased as cells differentiate into mature erythroid cells. The increase in mRNA observed at a later stage of maturation is unlikely to have functional relevance as erythroid cells expel their nucleus as part of their terminal differentiation, and RUNX3 function and localisation is nuclear. Human RNA-seq data demonstrated a similar trend, with erythroid cells having the lowest expression of RUNX3 compared to HSPC and compared to cell types from other lineages. We next examined the consequences of RUNX3 overexpression as a single abnormality on erythroid development. In early erythropoiesis, erythroid committed progenitors require SCF but not EPO for their proliferation^27^. Phenotypically, EPO independent erythroblast maturation is characterised by downregulation of CD34 on their cell surface while the thrombospondin receptor (CD36) is upregulated when cells commit to the erythroid lineage. We found that RUNX3 overexpression imposed a reduction in erythroid growth which was accompanied by a delayed upregulation of CD36 compared to controls suggesting a suppression of early human erythroid development by RUNX3.

EPO is absolutely required for the survival and proliferation of late erythroid progenitor cells and for their terminal differentiation^27^. In the presence of EPO, the erythroid marker GPA is upregulated, concomitant with a gradual downregulation of CD36^25^ and also a decrease in cell size^27^. During the EPO dependent phase of growth we found that RUNX3 overexpression promoted proliferation and impaired differentiation, evidenced by reduced upregulation of GPA and increased cell size compared to controls. Assessment of morphology supported the flow cytometric analysis of impaired differentiation. Previous studies have implicated RUNX3 in haematopoietic development using non-human models^12,15,28^. Recently, RUNX3 was identified as a key determinant of erythroid-myeloid lineage balance in the bone marrow and its expression was involved in the development of ageing-associated anaemias in humans^20^. Consistent with an inhibition of differentiation we also found evidence that RUNX3 overexpression increased colony formation. Interestingly, RUNX3 has been implicated in iron metabolism of the liver through regulation of BMP and TGF-β signallng^29^. In addition, a new role for TGF-β ligands in erythropoiesis has been discovered^30^ where the SMAD2/3 pathway is activated leading to increased cell proliferation of early (EPO-dependent) erythroid cells. Erythropoiesis and iron metabolism are intrinsically linked and RUNX3 maybe mediating this process. Further, our data parallels RUNX1-ETO-induced disruption of erythroid development and increased self-renewal of human HSPC^18^. RUNX1-ETO is known for retaining the Runt domain region of RUNX1 present in all RUNX proteins^31^, and therefore overexpression of RUNX3 and RUNX1-ETO could target similar processes in these cells. In the haematopoietic system, *RUNX1* expression is lost during erythroid development similarly to that of *RUNX3*^32,33^, and RUNX3-mediated repression of RUNX1 has been previously reported in different haematopoietic cells^16,34,35^. Considering the dysregulation of RUNX1 target genes by RUNX1-ETO^36^, overexpressing RUNX3 in HSPC could have similar effects leading to the inhibition of normal human erythropoiesis.

To assess the importance of RUNX3 expression during human erythroid development, its endogenous levels of expression were reduced using targeted shRNA. While this had little impact on development, RUNX3 KD efficiency was at best ~ 50% for any of the shRNA constructs employed in this study, hence we are unable to conclude that RUNX3 does not have a non-redundant role in human erythroid development. Functional redundancy between RUNX1 and RUNX3 could however rescue RUNX3 KD cells, as RUNX3 expression was previously shown to overlap that of RUNX1 in the haematopoietic system^37^ where combined RUNX1/RUNX3 knockout blocked murine erythropoiesis at early stages of development^15^. Under clonal conditions, KD of RUNX3 significantly inhibited erythroid colony formation indicative of a role in maintaining survival of erythroid progenitor cells. In support of this, conditional *RUNX3* knockout in aged mice was previously shown to significantly reduce the erythroid compartment^12^ and a similar inhibition of erythroid colony formation was recently shown in RUNX3 KD human HSPC^20^. This study suggested that RUNX3 has an important role in the maintenance of bone marrow lineage balance as RUNX3 KD selectively reduced the megakaryocyte-erythroid compartment along with myeloid skewing similar to ageing^20^. Phenotypic data obtained here contrast with recently published data by Balogh et al. suggesting that RUNX3 KD inhibits erythroid differentiation of human HSPC based on inhibition of GPA expression at a single timepoint after 3 days of culture^20^. We observed only a transient delay in GPA expression with all other differentiation endpoints being insignificantly altered. The reasons for contrasting results could be explained using different shRNA constructs targeting distinct regions of the *RUNX3* sequence, as well as different experimental designs.

In summary, RUNX3 expression decreases with erythroid maturation in human HSPC, and its ectopic expression leads to an impairment of normal erythroid differentiation and increased self-renewal. Taken together, this study demonstrates that the downregulation of RUNX3 observed in normal human erythropoiesis is important in promoting the terminal stages of erythroid development and may further our understanding of the role of this transcription factor in haematological disorders.

## Methods

### Plasmids and generation of retro- and lentivirus

A retroviral vector co-expressing RUNX3 and *Discosoma* sp. red fluorescent protein (DsRed) was generated by directional cloning of *RUNX3* (NM_001031680.2) into *Bam*H1/*Eco*R1 sites of a PINCO vector modified to express DsRed^38^. PINCO DsRed vector lacking *RUNX3* cDNA was used as control. Short hairpin RNA (shRNA) vectors co-expressing green fluorescent protein (GFP) were purchased from VectorBuilder (Guangzhou, China) (Supplemental Materials and Methods). RUNX3 shRNA vectors were selected using the Genetic Perturbation Platform (https://portals.broadinstitute.org/gpp/public/) according to their specificity score and match to RUNX3 CDS. Retro and lentivirus were subsequently generated by transient transfection of Phoenix or HEK293 packaging cells, respectively, using Lipofectamine 3000 (Fisher Scientific, Loughborough, UK) according to manufacturer’s instructions.

### Generation of control and RUNX3 expressing/knockdown human erythroid progenitor cells

Normal human CD34^+^ HSPC were isolated, cultured and transduced with unconcentrated retro/lentivirus as previously described (Supplemental Materials and Methods)^18,39^. For overexpression, cultures were transduced through two separate rounds (days) of infection. Lentivirus transduction underwent one round of infection to limit toxicity^40^. For each assay described, 3 or more independent cord blood samples were used. To aid the analysis of transduced viable erythroid committed cells, day 3 cultures were stained with CD13-allophycocyanin (APC) and further enriched for DsRed^+^CD13^low^ cells by FACS using a BD FACSAria III (BD Biosciences, Wokingham, UK), as previously described; cells were gated on the above parameters including FSC/SSC and doublet discrimination^18^. Sorted cells were subsequently used in colony assays (see “Colony assay”) or grown in bulk-liquid culture containing iron saturated human transferrin for growth and differentiation assessment by flow cytometry (see “Phenotypic, differentiation and morphology analysis”).

### Colony assay

Colony assays in liquid medium were performed as described previously^18^. Erythroid colony assays were performed on DsRed^+^CD13^low^ sorted HSPC on day 3 of culture by limiting dilution in 96-U plates (0.3 cells/well) in Iscove's Modified Dulbecco's Medium (IMDM; Fisher Scientific, Loughborough, UK) supplemented with IL-3, IL-6, SCF and EPO (BioLegend, London, UK) at 5 ng/mL or 2 U/mL for EPO and incubated at 37 °C with 5% CO_2_. Following 7 days of growth, individual erythroid colonies (> 50 cells) and clusters (> 5, < 50 cells) were counted and scored. BFU-e and CFU-e were not discriminated in these counts. To assess their self-renewal potential, colonies were harvested, replated at higher density (1 cell/well), and cultured for an additional week.

### Phenotypic, differentiation and morphological analysis

To assess erythroid cell growth and differentiation in bulk liquid culture, sorted HSPC were maintained in IMDM medium supplemented with IL-3, IL-6, and SCF at 5 ng/mL during the initial 10 days (EPO independent phase of development). On day 10 of culture, EPO at 2 U/mL was added to the growth medium, and the EPO dependent phase of development was monitored for additional 12 days. Transduced cultures were analysed by flow cytometry using a BD FACSCanto II at different time points using a panel of cell surface markers (Supplemental Materials and Methods and Supplemental Fig. S4) as previously described^18^. Morphology was assessed on day 20 of culture as previously described (Supplemental Materials and Methods)^18^.

### Validation of RUNX3 expression by western blot and qRT-PCR

Cytosolic and nuclear proteins were extracted using the Biovision Nuclear/Cytosol Fractionation Kit (Cambridge Bioscience, Cambridge, UK) following manufacturer’s instructions. Western blotting was performed as previously described (Supplemental Material and Methods)^41^ and RUNX3 protein expression was detected using a primary rabbit monoclonal antibody (D6E2, Cell Signaling Technologies, London, UK).

Total RNA was extracted from GFP^+^ sorted HSPC using the RNeasy Plus Mini Kit (Qiagen, Manchester, UK). RNA concentration and purity were assessed using a NanoDrop (Fisher Scientific UK Ltd, Loughborough, UK). *RUNX3* mRNA expression was determined using a TaqMan gene expression assay (Hs00231709_m1, Fisher Scientific UK Ltd, Loughborough, UK). *GAPDH* was used as reference gene (Hs02786624_g1). Gene expression was assessed using QuantStudio 5 Real-Time PCR System (Fisher Scientific UK Ltd, Loughborough, UK). Gene expression data was analysed using QuantStudio Design and Analysis software v1.5.1 by Thermo Fisher Scientific.

### Statistical and data analysis

Statistical analysis was performed using a paired sample t-test, or one-way ANOVA. Minitab 18 software (Minitab LLC, State College, Pennsylvania, USA) was used for all statistical analyses.

Gene expression data was obtained from GSE24759^42^. RNA-sequencing data from different human haematopoietic cells was obtained from the BLUEPRINT epigenome programme^43^.

### Ethics declaration

Human neonatal cord blood was obtained from the Maternity Unit of the University Hospital of Wales (Cardiff) in accordance with the 1964 Declaration of Helsinki. All methods were carried out in accordance with relevant guidelines and regulations. Informed consent was obtained from all subjects or, if subjects are under 18, from a parent and/or legal guardian. Use of cord blood was approved by South East Wales Local Research Ethics Committee 06/WSE03/6).

## Supplementary Information

Supplementary Information.

## Data Availability

Gene expression array data analysed in this study is available in the Gene Expression Omnibus (GEO) repository with the accession number GSE24759. Additional human RNA-seq data analysed in this study was obtained from the BLUEPRINT epigenome programme. All other data generated or analysed during this study are included in this published article (and its Supplementary Information files).
