# Supplementary material for: RUNX3 overexpression inhibits normal human erythroid development
Source: Sci Rep. 2022 Jan 24;12:1243. doi: 10.1038/s41598-022-05371-z (PMC8786893; doi:10.1038/s41598-022-05371-z)
Supplement: Supplementary file 1 — Supplementary Information. [file 41598_2022_5371_MOESM1_ESM.docx]

# Supplementary Information

**Article title**: RUNX3 overexpression inhibits normal human erythroid development

**Authors/Affiliations:** Ana Catarina Menezes,^1^ Christabel Dixon,^1^ Anna Scholz,^1^ Rachael Nicholson,^1^ Adam Leckenby,^1^ Aleksandra Azevedo,^1^ Sarah Baker,^1,2^ Amanda F Gilkes,^1,2^ Sara Davies,^1^ Richard L. Darley^1^ and Alex Tonks^1^*

^1^Department of Haematology, Division of Cancer & Genetics, School of Medicine, Cardiff University, Cardiff, CF14 4XN, U.K.

^2^Cardiff Experimental and Cancer Medicine Centre (ECMC), School of Medicine, Cardiff University, Cardiff, CF14 4XN, U.K.

# Supplemental materials and methods

## Isolation, infection and culture of human haematopoietic stem and progenitor cells

Human neonatal cord blood was obtained from the Maternity Unit of the University Hospital of Wales (Cardiff) in accordance with the 1964 Declaration of Helsinki. Normal human haematopoietic stem progenitor cells (HSPC) were isolated, cultured and transduced with retro/lentivirus as previously described.[1] Briefly, HSPC were infected with unconcentrated retro- or lentivirus by centrifugation for 120 minutes at 2200 *x g* and room temperature (RT) in 24-well plates pre-coated with 30 µg/mL RetroNectin (Takara, Paris, France).[2] Following centrifugation, the retroviral supernatant was removed and HSPC were added to the wells (70-140,000 cells/well). The infection procedure was repeated on the following day to improve the transduction efficiency for retroviral transduction. Following infection (day 3 of culture), cells were incubated at RT with CD13-allophycocyanin (APC), and transduced erythroid cells (DsRed^+^CD13^low^) were enriched by FACS using a BD FACSAria III (BD Biosciences, Wokingham, UK). Cells were maintained in Iscove's Modified Dulbecco's Medium (IMDM; Fisher Scientific, Loughborough, UK) containing 20% *v/v* FBS (Biosera Europe, France), 1% *v/v* BSA (Biosera Europe, France), human transferrin (30 mg/mL; Merck Life Science, Gillingham, UK), β-ME (9 mM; Merck Life Science, Gillingham, UK), gentamicin (20 µg/mL; Fisher Scientific, Loughborough, UK) and supplemented with 5 ng/mL of IL-3, IL-6 and SCF (BioLegend, London, UK). On day 10 of culture, erythropoietin (EPO; 2 U/mL; BioLegend, London, UK) was added and cultured for a further twelve days.

For overexpression experiments, three cultures were generated: mock, control (PINCO expressing DsRed alone) and RUNX3 co-expressing DsRed. For knockdown (KD) studies: mock, shRNA control GFP and 3 different shRNA targeting RUNX3 and co-expressing GFP were generated (see below).

## Source and structure of plasmids

A retroviral vector co-expressing RUNX3 and DsRed was generated by directional cloning of *RUNX3* (NM_001031680.2) into *Bam*H1/*Eco*R1 sites of PINCO vector. The source of plasmids used for both RUNX3 overexpression and KD studies in HSPC are listed in Table S1. Representation of plasmid maps for PINCO RUNX3 DsRed vector and RUNX3 shRNA GFP vector are shown in Figure S1.

***Table S1. Summary of retro- and lentiviral systems used for overexpression and KD studies.***

All vectors include the *ampicillin resistance* gene. Puro – Puromycin; NM Number – National Center for Biotechnology Information (NCBI) reference sequence (RefSeq) database transcript assession number; TRCN Number – The RNAi Consortium shRNA Clone ID number. PINCO was a kind gift from gift of Pier Pelicci, European Institute of Oncology, Milan, Italy.[3] RUNX3 shRNA KD vectors were purchased from VectorBuilder (Guangzhou, China).

| **Plasmid** | **Gene/Target sequence** | **Selectable marker** |
| --- | --- | --- |
| PINCO | Empty | DsRed |
| PINCO | RUNX3 [[NM_001031680.2](https://www.ncbi.nlm.nih.gov/nuccore/NM_001031680.2)] | DsRed |
| pLV | Scramble (control) shRNA | GFP/Puro |
| pLV | RUNX3 shRNA 1 [[TRCN0000235676](https://portals.broadinstitute.org/gpp/public/clone/details?cloneId=TRCN0000235676)]  GTTCAACGACCTTCGCTTCGT | GFP/Puro |
| pLV | RUNX3 shRNA 2 [[TRCN0000235675](https://portals.broadinstitute.org/gpp/public/clone/details?cloneId=TRCN0000235675)]  ACCACCTCTACTACGGGACAT | GFP/Puro |
| pLV | RUNX3 shRNA 3 [[TRCN0000235672](https://portals.broadinstitute.org/gpp/public/clone/details?cloneId=TRCN0000235672)]  TGGCAGGCAATGACGAGAACT | GFP/Puro |

## Phenotypic and differentiation analysis by flow cytometry

Transduced cultures were analysed by flow cytometry at different time points using a panel of cell surface markers (Table S2) as previously described.[1] CD13-APC in combination with CD36-biotin were used for lineage discrimination; Streptavidin PerCP-Cy5.5 was used as a second-step detection reagent. In addition, cells were incubated with one of the following Pacific Blue (PB)-labelled differentiation markers: glycophorin A (GPA) and CD34 (BioLegend, London, UK). All incubations were performed at 4ºC and reactions were controlled with the appropriate isotype-matched irrelevant antibody. Reagent concentrations were as recommended by the manufacturer.

***Table S2. Summary of antibodies used in flow cytometry.***

NA – Not applicable; Cambridge Bio – Cambridge Biosciences; CST – Cell Signaling Technology; SCBT – Santa Cruz Biotechnology.

| **Antibody** | **Clone** | **Supplier** |
| --- | --- | --- |
| Anti-Human CD13-APC | WM15 | BioLegend, London, UK |
| Anti-Human CD34-PB | 581 | BioLegend, London, UK |
| Anti-Human CD36-Biotin | NA | Cambridge Bio., Cambridge, UK |
| Anti-Human CD235a-PB | HI264 | BioLegend, London, UK |
| Mouse IgG1-PB | MOPC-21 | BioLegend, London, UK |
| PerCP-Cy™5.5 Streptavidin | NA | BD Biosciences, Wokingham, UK |

## Morphology

Approximately 30,000 cells were centrifuged (using Cytospin 3, 60 *x g* for 5 minutes) in a pre-assembled cytospin sample chamber with glass slide. Slides were stained with May–Grünwald–Giemsa for morphology examination and scanned using Zeiss Axioscan Z1 slide scanner (Carl Zeiss, Cambridge, UK) at 20X magnification. Differential counts were performed using Zen Lite software (Carl Zeiss, Cambridge, UK) to determine the number of cells in separate developmental stages. Erythroid cells in an early, intermediate, and late phase of development were defined as proerythroblasts, erythroblasts and normoblasts, respectively.

## Flow Cytometry

Flow cytometry data was acquired using a BD FACSCanto II (BD Biosciences, Wokingham, UK). The threshold for GFP/DsRed positivity was determined using identically treated mock transduced cultures. At least 20,000 events were recorded for each sample at a medium flow rate. Data were analysed using FCS Express v6 (De Novo Software, Pasadena, CA, USA). Debris and myeloid committed cells were excluded from all analyses based on light scatter and CD13 positivity, respectively.

## Western Blot

Cytosolic and nuclear proteins were extracted from from 5*x*10^6^ HSPC using the Biovision Nuclear/Cytosol Fractionation Kit (Cambridge Bioscience, Cambridge, UK). Briefly, 5*x*10^6^ cells were pelleted and washed, followed by sequential incubations with extraction buffers. Cytosol fractions were separated, and nuclear proteins were extracted using triethylammonium bicarbonate buffer (TEAB). Bradford protein assay was performed by measuring the absorbance of Bradford’s reagent solution (Sigma Aldrich, Dorset, UK) at 595 nm.

SDS-PAGE was performed as previously described[4] using the NuPAGE electrophoresis system (Fisher Scientific UK Ltd, Loughborough, UK). Detection of RUNX3 protein expression was determined using a primary rabbit monoclonal antibody (D6E2, Cell Signaling, London, UK) in conjunction with an anti-rabbit HRP Amersham ECL Advance Western Blotting Detection Kit (Cytiva, Little Chalfont, UK) according to the manufacturer’s instruction. GAPDH (6C5, SCBT, Heidlberg, Germany) and Histone 1 (AE-4, Bio-Rad, Hertfordshire, UK) protein expression was assessed for equal loading purposes. Densitometry was performed using ImageJ v1.8 software (<https://imagej.nih.gov/ij/>) by plotting a histogram of peak intensity for each band. The peak area was used as an arbitrary intensity value to estimate the fold changes in protein expression. Data was corrected for loading (GAPDH/Histone 1 expression) and normalised against control cells.

# Supplementary Figures

**a**


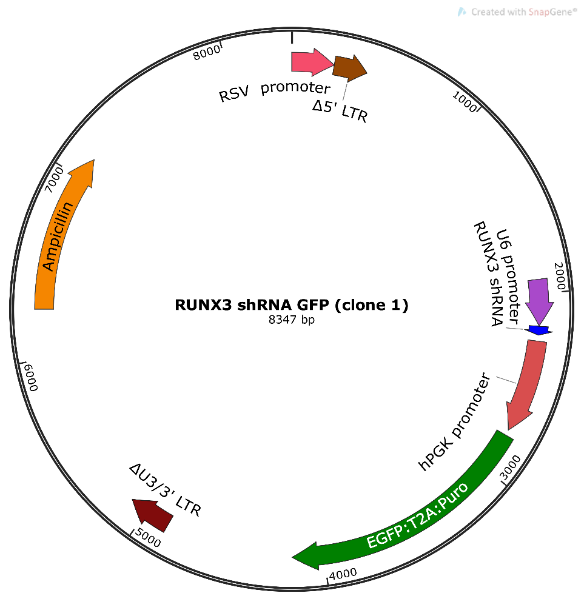


**b**

Figure S1. Plasmid DNA vectors used to overexpress and KD RUNX3 in human HSPC.

**(a)** Representation of PINCO RUNX3 DsRed vector created using SnapGene 5.2 software (GSL Biotech LLC, USA). Long terminal repeat (LTR) driving RUNX3 and cytomegalovirus promoter driving DsRed. **(b)** Representation of RUNX3 shRNA GFP vector for shRNA 1 (TRCN0000235675). This vector includes the *ampicillin resistance* gene; Rous sarcoma virus (RSV) promoter; LTR; U6 type III RNA polymerase promoter; the RUNX3 shRNA sequence; human phosphoglycerate kinase (hPGK) promoter; and EGFP:T2A:Puro sequence that confers both green fluorescence and resistance to puromycin.


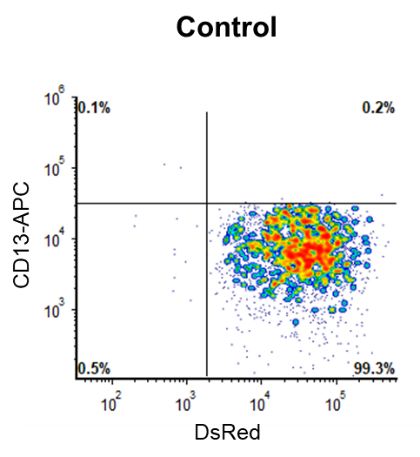

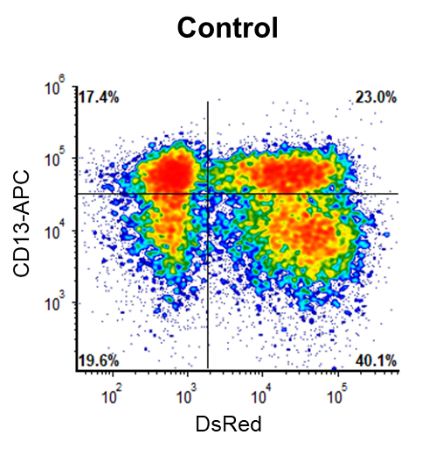


**Control post-sort**

**Control pre-sort**

**a**


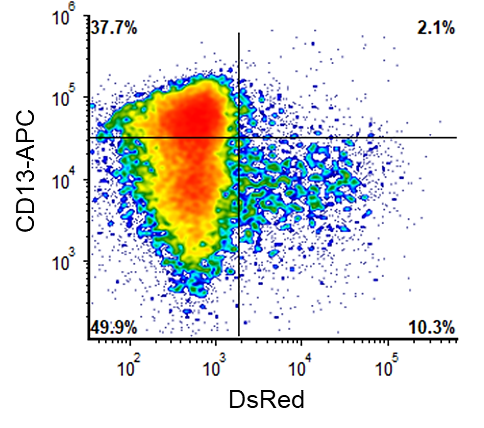


**b**

**RUNX3 pre-sort**

**RUNX3 post-sort**


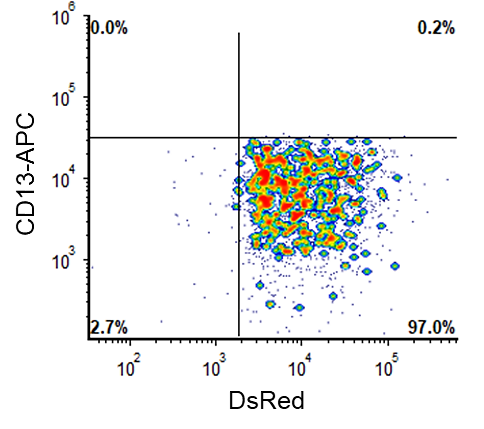


**c**

Figure S2. Sorting and lineage gating strategy of control and RUNX3 erythroid committed cells.

**(a)** Representative bivariate density plots of CD13 expression *vs* DsRed in (upper panels) control pre- and post-FACS (lower panels) RUNX pre- and post-FACS for CD13^low^DsRed^+^ (day 3 of culture). Quadrants delimit sorting conditions. **(b)** Example bivariate density plots of control and RUNX3 FACS cultures labelled with the lineage discriminators markers CD13 and CD36 at day 6 of early erythroid differentiation in the EPO independent phase of development.


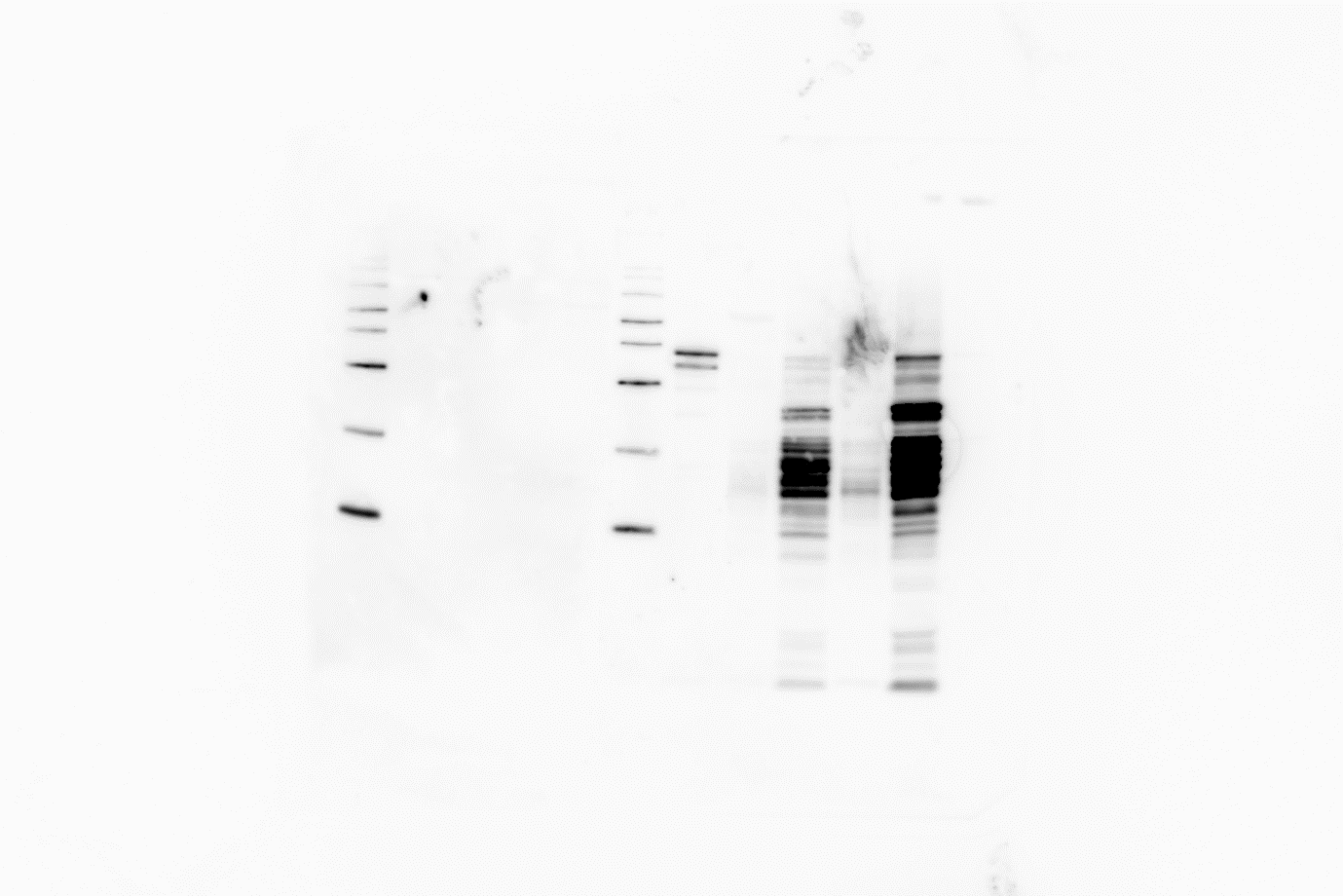


Control

RUNX3

PC

C

N

C

N

**a**

80kDa

60kDa

RUNX3

40kDa

30kDa

20kDa

**c**

**b**

Control

RUNX3

PC

C

N

C

N

Control

RUNX3

PC

C

N

C

N


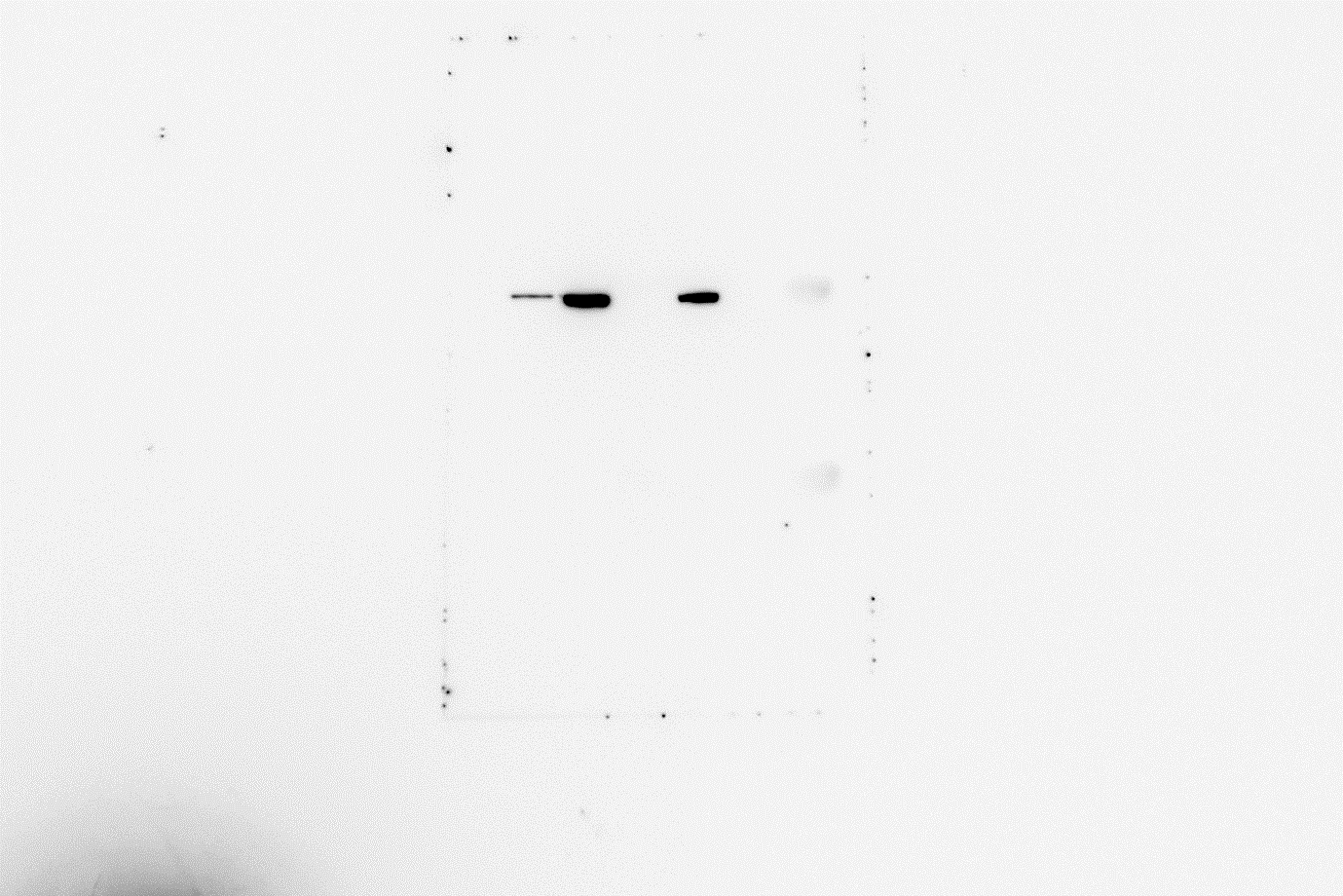


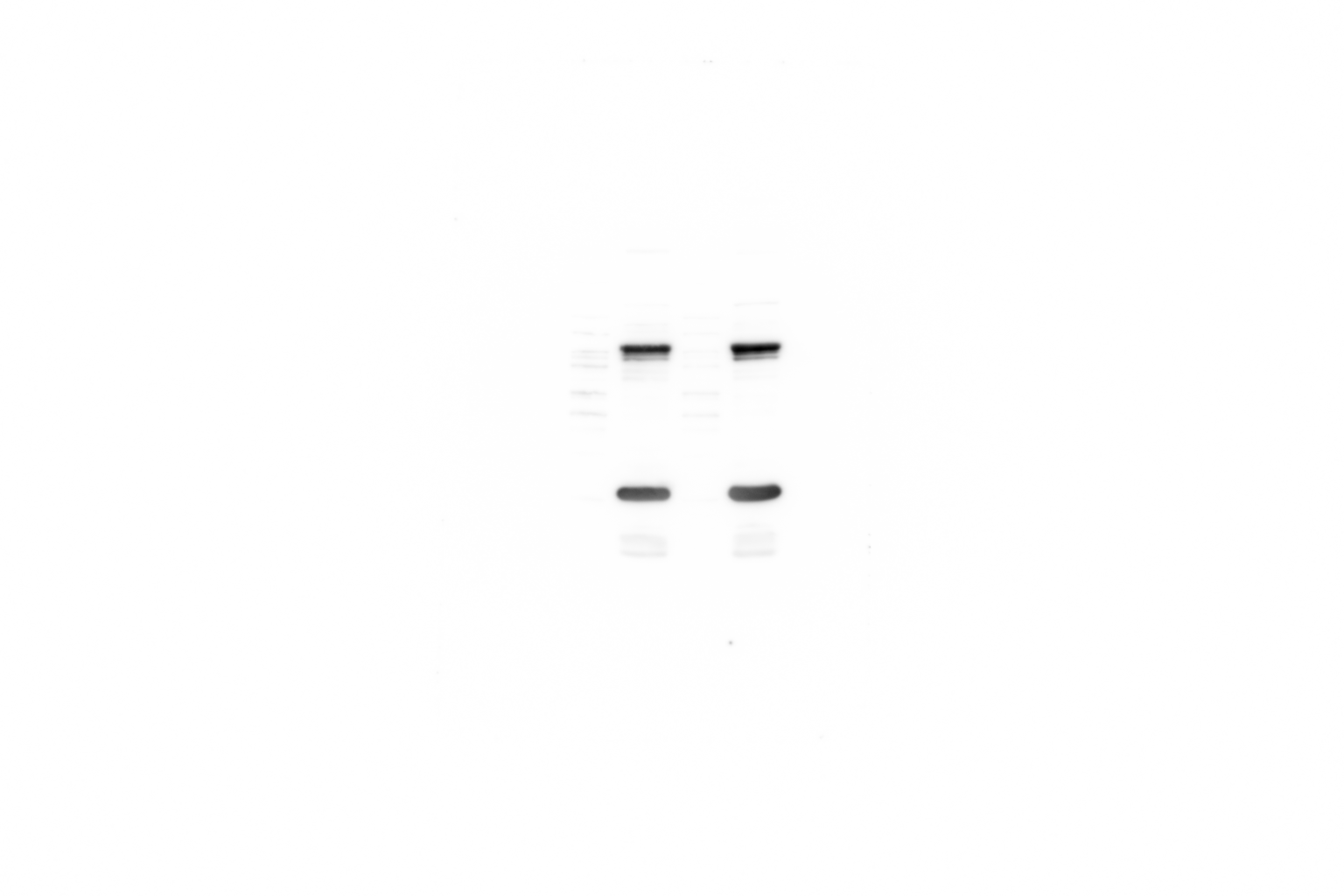


GAPDH

Histone H1

Figure S3. Full length western blots showing RUNX3 protein levels in the cytosol and nucleus of CD34^+^ HSPC

**(a)** Full length western blot showing RUNX3 protein levels in the cytosol and nucleus of control and RUNX3 CD34^+^ HSPC (day 6 of culture). Phoenix packaging cells overexpressing RUNX3 were used as a positive control. **(b)** Full length western blots showing reprobing of the above membrane for Histone H1 and GADPH (which were used as loading controls). PC – RUNX3 Positive control; C – Cytosol; N – Nucleus.

**Figure S4. Gating strategy used to follow the erythroid differentiation of HSPC by flow cytometry.**

Representative density plots and flow cytometry histograms of cells on day 6 of differentiation. Non-debris – Gate used to exclude all debris from the analysis. CD36^+^ – Gate to analyse the differentiation of erythroid committed cells CD13^–^CD36^+^ IgG-Pacific Blue HSPC cells – gray.


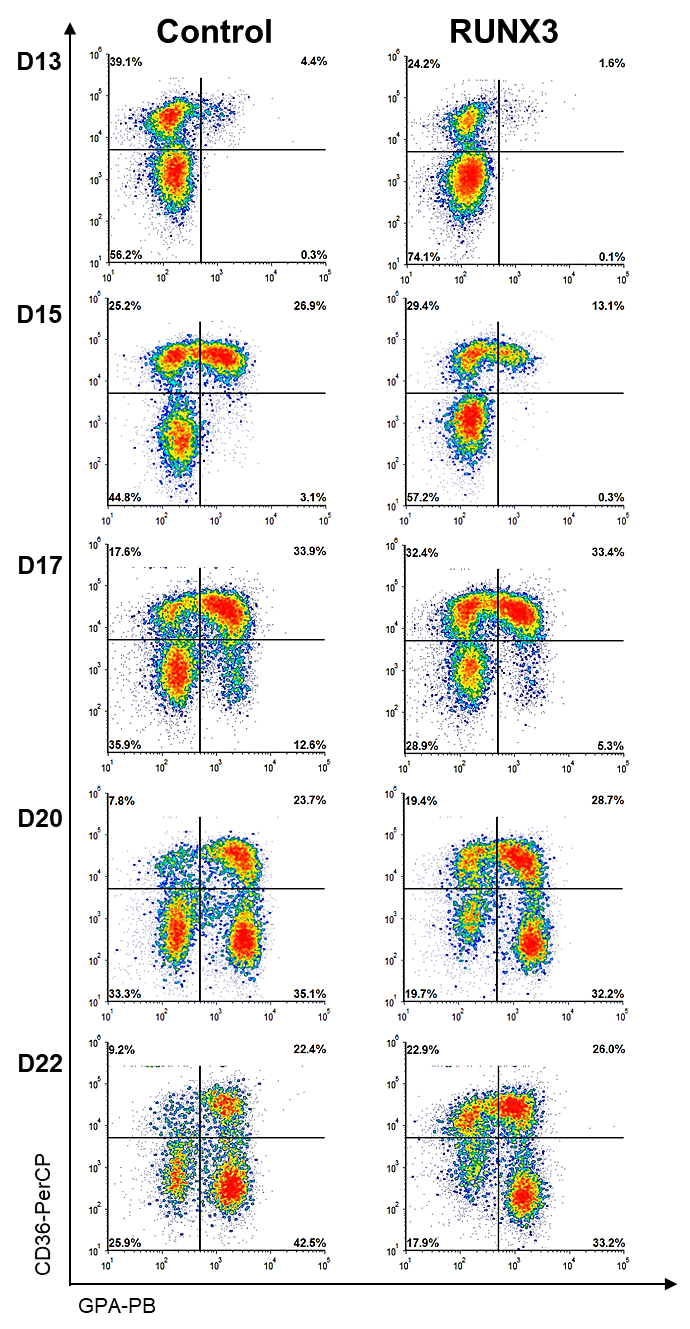


**Figure S5. RUNX3 overexpression promotes proliferation and supresses GPA expression during the EPO dependent phase of differentiation.**

Representative bivariate density plots of CD36 expression *vs* GPA in control and RUNX3 cultures during the EPO dependent phase of development (day 13-22).

**b**

**a**

**c**

**d**


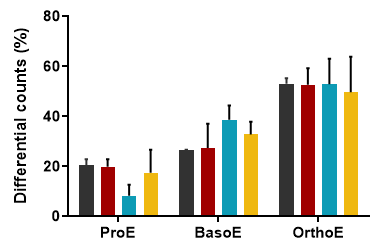


Figure S6. Knockdown of RUNX3 does not impair the erythroid differentiation of human HSPC.

**(a)** Example western blot of RUNX3 nuclear protein levels in OCI-AML-5 cells infected with a scramble shRNA (control) and different RUNX3 shRNA constructs. OCI-AML-5 parental cells were used as positive control and Histone 1 as a loading control. See Supplemental Figure S8 for full length blots. **(b)** Cumulative expansion of CD13^low^CD36^high^ erythroid progenitors in shRNA control and RUNX3 KD cultures during the EPO independent phase of growth (cells grown in the presence of SCF, IL-3 and IL-6). Data indicate mean ± 1SD (n≥4) and normalised against day 6. Significant differences were analysed by one-way ANOVA using Tukey’s test, **p=0.05.* **(c)** Summary plot showing cell size changes in size (FSC) for shRNA control and RUNX3 KD cultures during the EPO dependent phase of growth (cells grown in the presence of SCF, IL-3, IL-6 and EPO). Data indicate mean ± 1SD (n≥4). **(d)** Differential counts of all cultures with morphology categorised into ProE (proerythroblasts), BasoE (basophilic erythroblasts) and OrthoE (orthochromatic erythroblasts) on day 20 of differentiation during the EPO dependent phase of development. Data indicate mean ± 1SD (n≥3).

**a**

**b**

Figure S7. Knockdown of RUNX3 does not impair self-renewal of human HSPC.

Growth medium was supplemented with SCF, IL-3, IL-6, and EPO. **(a)** Self-renewal potential assessed by a single replating round of shRNA control and RUNX3 KD cultures in liquid culture. Data indicate mean ± 1SD (n≥3). **(b)** Erythroid cluster forming efficiency of shRNA control and RUNX3 KD cultures after 7 days of growth in liquid culture. Data indicate mean ± 1SD (n≥4).

**a**

Xp Ladder

Parental

shRNA Control

shRNA 1

shRNA 2

shRNA 3





80kDa

40kDa

60kDa

RUNX3

30kDa

20kDa

Parental

shRNA Control

shRNA 1

shRNA 2

shRNA 3

**b**





Histone H1

Figure S8. Full length blots showing Knockdown of RUNX3 in OCI-AML-5.

**(a)** Full length western blot showing RUNX3 nuclear protein levels in OCI-AML-5 cells infected with a scramble shRNA (control) and different RUNX3 shRNA constructs. (b) Full length western blots showing reprobing of the above membrane for Histone H1 which was used as loading control.

## References

1 Tonks, A. *et al.* The AML1-ETO fusion gene promotes extensive self-renewal of human primary erythroid cells. *Blood* **101**, 624-632, doi:10.1182/blood-2002-06-1732 (2003).

2 Tonks, A. *et al.* Optimized retroviral transduction protocol which preserves the primitive subpopulation of human hematopoietic cells. *Biotechnol Prog* **21**, 953-958, doi:10.1021/bp0500314 (2005).

3 Grignani, F. *et al.* High-efficiency gene transfer and selection of human hematopoietic progenitor cells with a hybrid EBV/retroviral vector expressing the green fluorescence protein. *Cancer Res* **58**, 14-19 (1998).

4 Tonks, A. *et al.* Transcriptional dysregulation mediated by RUNX1-RUNX1T1 in normal human progenitor cells and in acute myeloid leukaemia. *Leukemia* **21**, 2495-2505, doi:10.1038/sj.leu.2404961 (2007).
